# Correction: Chen et al. Development and Comparison of Treatment Decision Tools for Glucocorticoid-Induced Osteoporosis. *Diagnostics* 2024, *14*, 452

**DOI:** 10.3390/diagnostics15222861

**Published:** 2025-11-12

**Authors:** Jia-Feng Chen, Shan-Fu Yu, Wen-Chan Chiu, Chi-Hua Ko, Chung-Yuan Hsu, Han-Ming Lai, Ying-Chou Chen, Yu-Jih Su, Hong-Yo Kang, Tien-Tsai Cheng

**Affiliations:** 1 Division of Rheumatology, Allergy, and Immunology, Department of Internal Medicine, College of Medicine, Kaohsiung Chang Gung Memorial Hospital, Chang Gung University, Kaohsiung 833, Taiwan; 2 Graduate Institute of Clinical Medical Sciences, College of Medicine, Chang Gung University, Kaohsiung 833, Taiwan; 3 Center for Menopause and Reproductive Medicine Research, Department of Obstetrics and Gynecology, College of Medicine, Kaohsiung Chang Gung Memorial Hospital, Chang Gung University, Kaohsiung 833, Taiwan; 4 Division of Endocrinology and Metabolism, Department of Internal Medicine, College of Medicine, Kaohsiung Chang Gung Memorial Hospital, Chang Gung University, Kaohsiung 833, Taiwan; 5 Department of Biological Sciences, National Sun Yat-Sen University, Kaohsiung 804, Taiwan

The authors are issuing a corrigendum for their article [1] to correct information within the Institutional Review Board Statement and the study period. These inaccuracies were the result of an administrative oversight.

## Institutional Review Board Statement Correction

The IRB protocol code was erroneously stated. The correct IRB protocol code is 104-3530B, with an approval date of 13 July 2015. The corrected Institutional Review Board Statement appears below:

The study protocol was approved by the Regional Ethical Review Board of Chang Gung Memorial Hospital, Kaohsiung (protocol code: 104-3530B, date of approval: 13 July 2015). Data was collected from September 2015 through April 2021.

## Text Correction

Corrections have been made to the study period in the Abstract, Materials and Methods Section and Figure 1: The study period was stated incorrectly. The data for this registry were collected at Chang Gung Memorial Hospital, Kaohsiung, Taiwan, from September 2015 to April 2021.

A correction has been made to the Abstract:

We utilized registry data gathered at Chang Gung Memorial Hospital in Kaohsiung, Taiwan, between September 2015 and April 2021.

A correction has been made to the Materials and Methods, paragraph 1:

A registry was compiled at Chang Gung Memorial Hospital, Kaohsiung (CGMHK), Taiwan, between September 2015 and April 2021.

The corrected Figure 1 appears below:

The scientific integrity and conclusions of the study remain unaffected by this correction. The authors regret any confusion this may have caused. This correction was approved by the Academic Editor. The original publication has also been updated.

## Figures and Tables

**Figure 1:**
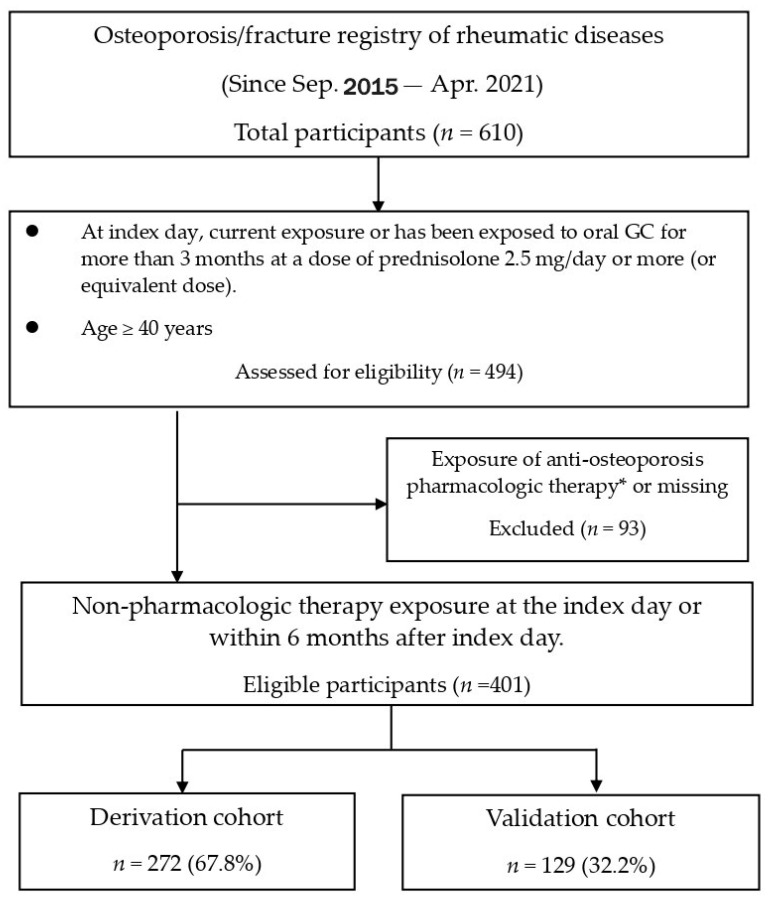
Disposition of participants. GC = glucocorticoid. * Exposure of therapy is defined as therapy exposure currently on the index day, or within the previous 6 months before the index day, or within the upcoming 6 months after the index day. Pharmacologic therapy included selective estrogen receptor modulators, oral or intravenous bisphosphonates, denosumab, and teriparatide.
