# Supplementary material for: Development and Comparison of Treatment Decision Tools for Glucocorticoid-Induced Osteoporosis
Source: Diagnostics (Basel). 2024 Feb 19;14(4):452. doi: 10.3390/diagnostics14040452 (PMC10887529; doi:10.3390/diagnostics14040452)
Supplement: Supplementary file 1 [file diagnostics-14-00452-s001.zip › diagnostics-2870886-supplementary.pdf]

Supplementary Online materials

Development and Comparison of Treatment Decision Tools for Glucocorticoid-Induced Osteoporosis

Supplementary Table S1. Treatment recommendations of IOF-ECTS and ACR.

Supplementary Table S2. Characteristics of all study participants.

Supplementary Table S3. Demographic data of study participants in derivation and validation cohorts.

Supplementary Table S4. Univariate logistic regression in the derivation set.

Supplementary Figure S1. Selection of candidate predictors by LASSO logistic regression method.

Supplementary Table S5. Model development by adding candidate predictors in sequence (A), discriminative (B) and calibration plot (C) of selected model

Supplemental References

Supplementary Table S1. Treatment recommendations of IOF-ECTS and ACR

Society	Year	Treatment case finding strategy	Reference
**IOF-ECTS**	2012	1. Prior history of OP fracture **OR**<br>2. Age ≥70 years **OR**<br>3. Prednisolone ≥7.5 mg/day **OR**<br>4. GC-adjusted FRAX above intervention threshold	[1]
**ACR**	2017<br>2022 update	Fracture risk stratified as “moderate, high or very high” (age≥40), described as<br>1. Prior history of OP fractures **OR**<br>2. BMD T-score between -1 and -2.4 **OR**<br>3. BMD T-score ≤-2.5<br>4. GC-adjusted FRAX 10-year risk for major osteoporotic fracture ≥ 10% **OR**<br>5. GC-adjusted FRAX 10-year risk for hip fracture ≥ 1% **OR**<br>6. 30mg/day or cumulative GC doses ≥ 5 grams/year	[2,3]

IOF-ECTS, International Osteoporosis Foundation-European Calcified Tissue Society; ACR, American College of Rheumatology; GC, glucocorticoid; OP, osteoporosis; MP, menopause;
[&] ≥ 7.5 mg/prednisolone or equivalent/day

Supplementary Table S2. Characteristics of all study participants.

Variables	All participants N = 401
Age (years)	57.5 ± 8.3
Female, n (%)	345 (86.0)
Body weight (kg)	58.2 ± 11.0
Body height (cm)	156.4 ± 6.7
Body mass index (kg/cm^2)	23.8 ± 3.9
New fracture +, n (%)	78 (19.5)
Fracture risk factors	
Rheumatic diseases, n (%)	
RA	324 (80.8)
SLE	40 (10.0)
Sjögren's syndrome	9 (2.2)
Others*	28 (7.0)
Previous fracture +, n (%)	90 (22.4)
2nd osteoporosis +, n (%)	20 (5.0)
Glucocorticoid	
Daily dose	4.2 ± 1.7
Exposure duration, n (%)	
≥3 months	401 (100)
≥6 months	374 (93.3)
≥12 months	364 (90.8)
≥2 years	323 (80.5)
≥3 years	289 (72.1)
Accumulative dose (mg)	
In 3 months	378.3 ± 249.6
In 6 months	775.6± 417.3
Parent fractured hip +, n (%)	28 (7.0)
Osteoporosis +, n (%)	115 (28.7)
BMD (g/cm^2)	
FN§	0.636 ± 0.111
TH©	0.797 ± 0.140
L1-4 ®	0.868 ± 0.166
Current smoking +, n (%)	23 (5.7)
Alcohol +, n (%)	6 (1.5)
Previous fall +, n (%)	75 (18.7)
FRAX	
Major fracture (%)	16.9 ± 12.3
Hip fracture (%)	7.0 ± 8.6

RA = Rheumatoid arthritis, SLE = systemic lupus erythematous, * this item includes vasculitis, systemic sclerosis, allergy and inflammatory myopathy, BMD = Bone mineral density, FN = femoral neck, TH = total hip, L1-4 = lumbar spine L1-L4. FRAX = Fracture Risk Assessment Tool.

Supplementary Table S3. Demographic data of study participants in derivation and validation cohorts.

Variables	All participants N= 401	Derivation cohort n=272 (67.8)	Validation cohort n=129 (32.2)	p-value
Age (years)	57.5 ± 8.3	57.4 ± 8.4	57.6 ± 8.1	0.86
Female, n (%)	345 (86.0)	240 (88.2)	105 (81.4)	0.07
Body weight (kgs)	58.2 ± 11.0	58.5 ± 11.6	57.7 ± 9.7	0.48
Body height (cm)	156.4 ± 6.7	156.2 ± 6.8	156.9 ± 6.4	0.32
Body mass index (kgs/cm^2)	23.8 ± 3.9	23.9 ± 4.1	23.4 ± 3.3	0.19
New Fracture +, n(%)	78 (19.5)	49 (18.0)	29 (22.5)	0.29
Fracture risk factors				
Rheumatic diseases, n(%)				
RA	324 (80.8)	223 (82.0)	101 (78.3)	0.81
SLE	40 (10.0)	26 (9.6)	14 (10.9)	
Sjogren's syndrome	9 (2.2)	6 (2.2)	3 (2.3)	
Others	28 (7.0)	17 (6.3)	11 (8.5)	
Previous fracture +, n (%)	90 (22.4)	59 (21.7)	31 (24.0)	0.60
2nd Osteoporosis +, n (%)	20 (5.0)	12 (4.4)	8 (6.2)	0.44
Glucocorticoid				
Daily dose	4.2 ± 1.7	4.1 ± 1.7	4.2 ± 1.6	0.56
Exposure duration, n (%)				
≥ 3 months	401 (100)	272 (100)	129 (100)	-
≥ 6 months	374 (93.3)	257 (94.5)	117 (90.7)	0.16
≥ 12 months	364 (90.8)	248 (91.2)	116 (89.9)	0.69
≥ 2 yrs	323 (80.5)	219 (80.5)	104 (80.6)	0.98
≥ 3 yrs	289 (72.1)	194 (71.3)	95 (73.6)	0.63
Accumulative dose (mg)*				
3 months	378.3 ± 249.6	381.1 ± 272.0	372.3 ± 194.6	0.74
6 months	775.6± 417.3	778.7 ± 444.9	769.1 ± 353.5	0.83
Parent fractured hip +, n (%)	28 (7.0)	18 (6.6)	10 (7.8)	0.68
Osteoporosis +, n (%)	115 (28.7)	76 (27.9)	39 (30.2)	0.64
BMD (g/cm^2)				
FN	0.636 ± 0.111	0.639 ± 0.114	0.630 ± 0.104	0.44
TH	0.797 ± 0.140	0.802 ± 0.143	0.787 ± 0.132	0.33
L1-4	0.868 ± 0.166	0.864 ± 0.167	0.878 ± 0.163	0.43
Current smoking +, n (%)	23 (5.7)	14 (5.1)	9 (7.0)	0.46
Alcohol +, n (%)	6 (1.5)	3 (1.1)	3 (2.3)	0.35
Previous Fall +, n (%)	75 (18.7)	51 (18.8)	27 (20.9)	0.61
FRAX				
Major fracture (%)	16.9 ± 12.3	16.9 ± 12.4	17.0 ± 12.2	0.90
Hip fracture (%)	7.0 ± 8.6	7.0 ± 8.9	6.9 ± 7.9	0.88

RA = Rheumatoid arthritis, SLE = systemic lupus erythematous, * this item includes vasculitis, systemic sclerosis, allergy and inflammatory myopathy, BMD = Bone mineral density, FN = femoral neck, TH = total hip, L1-4 = lumbar spine L1-L4. FRAX = Fracture Risk Assessment Tool.

Supplementary Table S4. Univariate logistic regression in the derivation set.

Variables	comparison	B-Coefficient	OR (95% CI)	p-value
Age (years)	Per 1 year increase	0.05	1.05 (1.01-1.09)	0.02
Female, n (%)	male	0.28	1.32 (0.54-3.25)	0.55
Body weight (kgs)	Per 1 kg increase	0.01	1.01 (0.98-1.03)	0.53
Body height (cm)	Per 1 cm increase	-0.02	0.98 (0.94-1.03)	0.37
Body mass index (kgs/cm^2)	Per 1 unit increase	0.63	1.87 (1.00-3.53)	0.05
Previous fracture +, n (%)	No	1.06	2.90 (1.48-5.66)	0.002
Glucocorticoid				
Daily dose	Per 1 mg increase	0.49	1.64 (0.72-3.71)	0.24
Exposure duration, n (%)				
≥ 6 months	< 6 months	-0.54	0.58 (0.18-1.92)	0.38
≥ 12 months	<12 months	-0.20	0.82 (0.29-2.31)	0.71
≥ 2 yrs	<2 years	0.09	1.09 (0.49-2.42)	0.83
≥ 3 yrs	<3 years	0.13	1.14 (0.57-2.29)	0.71
Accumulative dose (mg)*				
3 months, ≥ 300mg	< 300 mg	0.22	1.25 (0.67-2.32)	0.48
6 months, ≥ 750mg	<750 mg	0.68	1.98 (1.02-3.84)	0.04
Parent fractured hip +, n (%)	No	0.28	1.33 (0.41-4.22)	0.63
Osteoporosis +, n (%)	No	0.83	2.29 (1.21-4.36)	0.01
Current smoking +, n (%)	No	0.64	1.89 (0.57-6.30)	0.30
Alcohol +, n (%)				
Previous Fall +, n (%)	No	0.56	1.76 (0.85-3.62)	0.13
FRAX				
Major fracture (%)	Per 1 unit increase	0.04	1.04 (1.02-1.07)	<0.001
Hip fracture (%)	Per 1 unit increase	0.05	1.06 (1.02-1.09)	0.001
Lab				
White blood cell (10^3/μl)	Per 1 unit increase	-0.01	0.99 (0.85-1.16)	0.89
Hemoglobin (g/dL)	Per 1 unit increase	-0.25	0.98 (0.77-1.24)	0.84
Platelet (10^3/μl)	Per 1 unit increase	0.001	1.00 (1.00-1.01)	0.65
BUN (mg/dL)	Per 1 unit increase	0.01	1.01 (0.95-1.08)	0.71
Creatinine (mg/dL)	Per 1 unit increase	-0.99	0.37 (0.05-2.80)	0.34
AST (U/L)	Per 1 unit increase	-0.005	1.00 (0.97-1.02)	0.66
ALT (U/L)	Per 1 unit increase	-0.003	1.00 (0.98-1.01)	0.66
Albumin (g/dL)	Per 1 unit increase	0.73	2.08 (0.56-7.77)	0.28
Calcium (mg/dL)	Per 1 unit increase	0.007	1.01 (0.42-2.42)	0.99
Phosphate (mg/dL)	Per 1 unit increase	0.004	1.00 (0.52-1.93)	0.99

Supplementary Figure S1. Selection of candidate predictors by LASSO logistic regression method.

A. LASSO coefficients of 7 candidate predictors against log (Lambda)

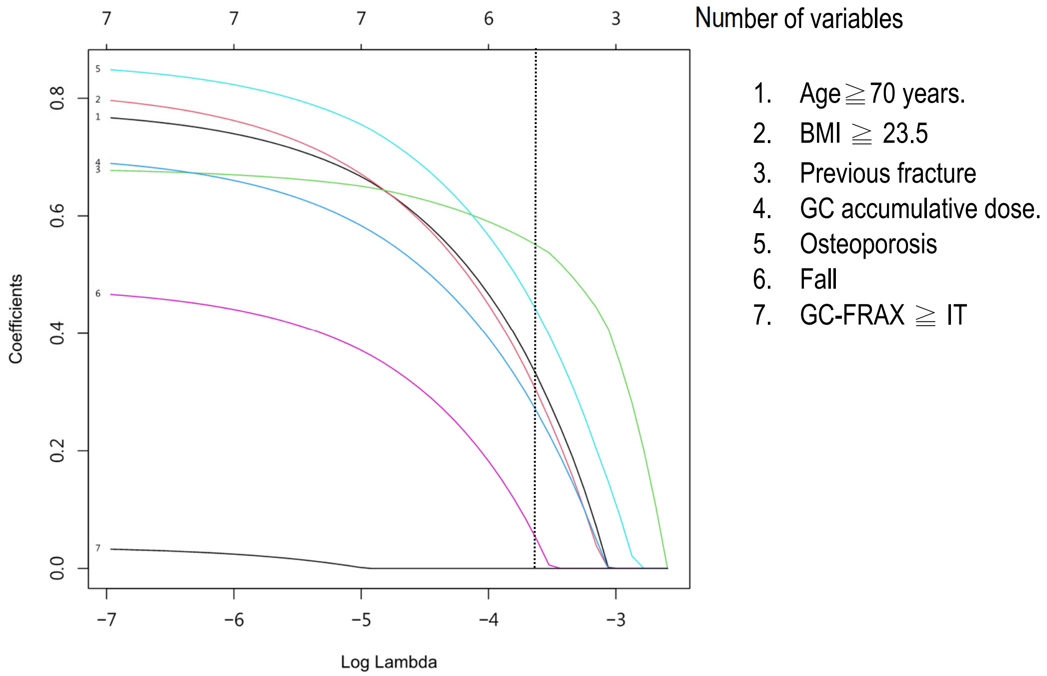

B. Binomial deviance curve versus log (lambda)

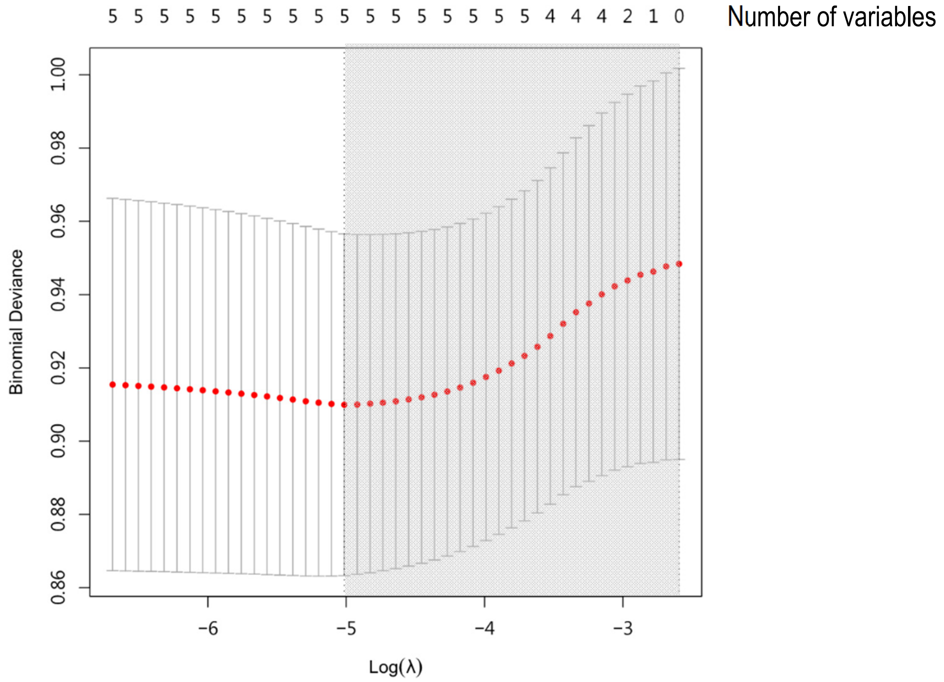

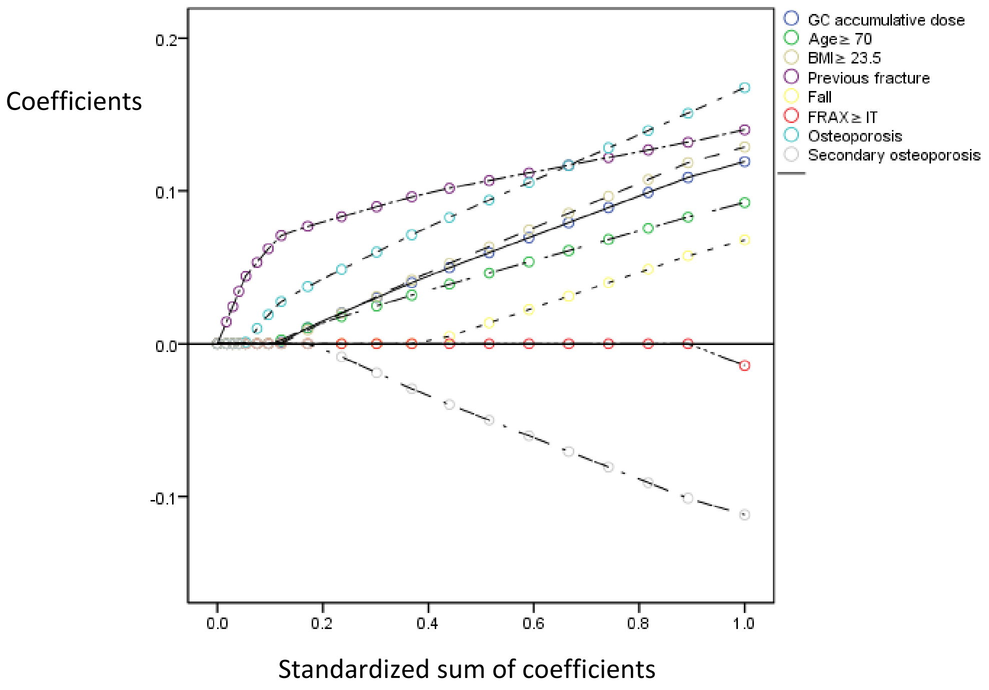
Coefficients
0.2
0.1
0.0
-0.1
0.0
0.2
0.4
0.6
0.8
1.0
Standardized sum of coefficients
GC accumulative dose
Age≥ 70
BMI≥ 23.5
Previous fracture
Fall
FRAX≥ IT
Osteoporosis
Secondary osteoporosis

Supplementary Table S5. Model development by adding candidate predictors in sequence (A), discriminative (B) and calibration plot (C) of selected model

(A) Model development

			ROC curve				Hosmer-Lemeshow test	
Test model	Predictors	Score range	AUC	Standard error	95% confidence interval	p-value	Chi-square (X^2)	p-value
A	Previous Fracture<br>+ Osteoporosis	0-2	0.65	0.05	0.56-0.74	0.001	0.07	0.97
B	Previous Fracture<br>+ Osteoporosis<br>+ BMI	0-3	0.68	0.04	0.60-0.76	<0.001	0.58	0.97
C	Previous Fracture<br>+ Osteoporosis<br>+ BMI<br>+ GC accumulative dose	0-4	0.70	0.04	0.62-0.78	<0.001	4.43	0.62
D	Previous Fracture<br>+ Osteoporosis<br>+BMI<br>+ GC accumulative dose<br>+ Age (x2)	0-6	0.71	0.04	0.64-0.79	<0.001	8.24	0.22
E	Previous Fracture<br>+ Osteoporosis<br>+BMI<br>+ GC accumulative dose<br>+ Age (x2)<br>+ Fall	0-7	0.73	0.04	0.65-0.80	<0.001	7.13	0.42
F	Previous Fracture<br>+ Osteoporosis<br>+BMI<br>+ GC accumulative dose<br>+ Age (x2)<br>+ Fall<br>+ FRAX ≥IT	0-8	0.71	0.04	0.64-0.79	<0.001	6.63	0.58

(B) Discriminative performance of model D

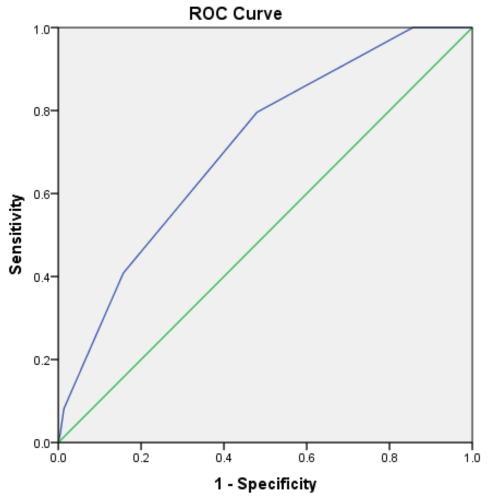

(C) Calibration plot of model D.

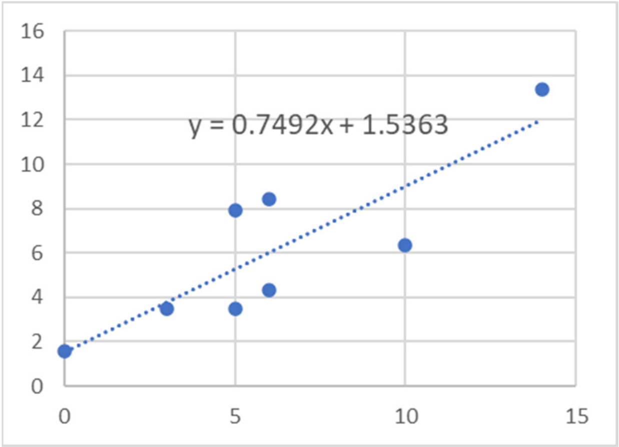

Reference

1. Lekamwasam, S.; Adachi, J.D.; Agnusdei, D.; Bilezikian, J.; Boonen, S.; Borgstrom, F.; Cooper, C.; Diez Perez, A.; Eastell, R.; Hofbauer, L.C.; et al. A framework for the development of guidelines for the management of glucocorticoid-induced osteoporosis. *Osteoporos Int* **2012**, *23*, 2257-2276, doi:10.1007/s00198-012-1958-1.
2. Buckley, L.; Guyatt, G.; Fink, H.A.; Cannon, M.; Grossman, J.; Hansen, K.E.; Humphrey, M.B.; Lane, N.E.; Magrey, M.; Miller, M.; et al. 2017 American College of Rheumatology Guideline for the Prevention and Treatment of Glucocorticoid-Induced Osteoporosis. *Arthritis Rheumatol* **2017**, *69*, 1521-1537, doi:10.1002/art.40137.
3. Humphrey, M.B.; Russell, L.; Danila, M.I.; Fink, H.A.; Guyatt, G.; Cannon, M.; Caplan, L.; Gore, S.; Grossman, J.; Hansen, K.E.; et al. 2022 American College of Rheumatology Guideline for the Prevention and Treatment of Glucocorticoid-Induced Osteoporosis. *Arthritis Care Res (Hoboken)* **2023**, *75*, 2405-2419, doi:10.1002/acr.25240.
